# Spectrum Sensing Method Based on Information Geometry and Deep Neural Network

**DOI:** 10.3390/e22010094

**Published:** 2020-01-12

**Authors:** Kaixuan Du, Pin Wan, Yonghua Wang, Xiongzhi Ai, Huang Chen

**Affiliations:** 1 School of Automation, Guangdong University of Technology, Guangzhou 510006, China; 2111704051@mail2.gdut.edu.cn (K.D.); wanpin2@163.com (P.W.); 17607011056@163.com (X.A.); gdutchenhuang@163.com (H.C.); 2 Hubei Key Laboratory of Intelligent Wireless Communications, South-Central University for Nationalities, Wuhan 430074, China; 3 State Key Laboratory of Management and Control for Complex Systems, Institute of Automation, Chinese Academy of Sciences, Beijing 100190, China

**Keywords:** spectrum sensing, information geometry, statistical manifold, geodesic distance, deep neural network

## Abstract

Due to the scarcity of radio spectrum resources and the growing demand, the use of spectrum sensing technology to improve the utilization of spectrum resources has become a hot research topic. In order to improve the utilization of spectrum resources, this paper proposes a spectrum sensing method that combines information geometry and deep learning. Firstly, the covariance matrix of the sensing signal is projected onto the statistical manifold. Each sensing signal can be regarded as a point on the manifold. Then, the geodesic distance between the signals is perceived as its statistical characteristics. Finally, deep neural network is used to classify the dataset composed of the geodesic distance. Simulation experiments show that the proposed spectrum sensing method based on deep neural network and information geometry has better performance in terms of sensing precision.

## 1. Introduction

Cognitive radio (CR) is a method to alleviate the allocation of spectrum resources in response to the shortage of spectrum resources [1]. Spectrum sensing is an important technology of cognitive radio. Classical spectrum sensing algorithms include energy detection, matched filter detection, and cyclostationary characteristic detection [2]. On the basis of classical spectrum sensing, a study proposed a goodness-of-fit test algorithm [3]. The goodness-of-fit test is to study the overall distribution of the sensing signal, so this algorithm shows better performance than the classical algorithm in terms of sensing accuracy. Traditional sensing methods and goodness-of-fit tests are single secondary user (SU) methods, which are easily interfered by propagation environment factors. Therefore, some scholars applied the cooperative spectrum sensing algorithm to avoid such interference [4,5,6]. A sensing method based on random matrix theory (RMT) is one of cooperative spectrum sensing methods. Based on the RMT theory, a study proposed a spectrum sensing algorithm based on the maximum eigenvalue detection (MED), but this algorithm only uses the single threshold detection of the largest eigenvalue to cause the detection performance [7]. The work in [8] proposed a maximum eigenvalue-energy detection (ME-ED) spectrum sensing algorithm. The algorithm uses the ratio of the maximum eigenvalue and the average energy of the sampled signal as a statistical feature. It compares the statistical feature with the threshold to determine whether the primary user (PU) signal exists. However, the ME-ED does not participate in the calculation of the minimum eigenvalue of the sensing signal, so the information of the sensing signal is not fully utilized. A study proposed the maximum and minimum eigenvalue (MME) spectrum sensing algorithm, which made full use of the sampling information [9]. However, if the sampling information is small, there is a large error in the acquisition of the eigenvalue, which affects the performance of the algorithm. The research work [10] proposed a spectrum sensing algorithm based on the difference between the maximum and minimum eigenvalues (DMM). The algorithm eliminates the noise component by the difference between the maximum and minimum eigenvalues, so that the same environment can show better performance.

The classical spectrum sensing methods often need to set the corresponding decision threshold according to the statistical characteristics of the eigenvalues. Next, we judge that the PU exists by the judgment criterion. When machine learning is used for classification, it has the advantages of high classification accuracy and strong learning ability. Therefore, more and more literature works have combined machine learning and spectrum sensing for classification. The work in [11] used the support vector machine method in machine learning to classify the training data. It did not need a decision threshold, but it improved the sensing accuracy. Some scholars [12] applied a combination of the k-means clustering algorithm and spectrum sensing in unsupervised learning. It achieved blind detection of the PU signal with low signal-to-noise ratio (SNR) and exhibited good sensing performance.

In this paper, the characteristics of the sensing signal matrix are extracted by way of information geometry, and the performance of spectrum sensing is improved from a new perspective. Information geometry is a set of theoretical systems developed from the study of probability distribution manifolds [13]. The concept of information geometry can be used to transform the detection problem on the spectrum into a geometric problem on the statistical manifold. The geometric method on the statistical manifold can be used to analyze the signal detection problem more intuitively [14]. In order to improve the sensing accuracy, a feature extraction method was introduced [15]. It used the geodesic distance between signals as a feature to detect signals, thus achieving the purpose of combining information geometry with spectrum sensing. The work in [16] was mainly to solve the influence of noise on the sensing precision in complex environments, and it proposed a sensing algorithm based on empirical mode decomposition (EMD) and information geometry. It used EMD to perform noise reduction processing and then used information geometry to perform feature extraction. A recent research work applied unsupervised learning to the classification of sensing signals [17]. This study combined information geometry and fuzzy C-means clustering algorithms to improve classification.

Deep learning has good migration learning ability, and its application improves the sensing performance in spectrum sensing. In [18], a blind spectrum sensing method based on deep learning was proposed. By comparing this method with an energy detector, good sensing performance under the low SNR was achieved. Xie et al. [19] proposed a spectrum sensing algorithm based on a deep learning convolutional neural network, which avoided the influence of the accuracy of the hypothetical statistical model on the detection results and improved the detection probability. Ke et al. [20] used a convolutional neural network (CNN) and the long short term memory (LSTM) methods for passive signal detection, improving signal detection performance in low SNR environments. The work in [21] proposed a hierarchical large scale neural network model, which was a hierarchical extension based on information geometry, thus improving the ability to process data.

In this paper, deep learning is applied to spectrum sensing, and the spectrum sensing method based on information geometry and deep neural network (IG-DNN) is proposed. Compared with the literature on spectrum sensing, which combines information geometry with unsupervised learning clustering algorithms, the innovation in this paper is a combination of deep learning algorithms and information geometry. The neural network algorithm in supervised learning is used to train the distance characteristics between the sampled signals to obtain a classifier. The method not only jumps out of the traditional spectrum sensing method, but also combines with deep learning to classify and thus improve the sensing effect.

The remainder of this paper is described as follows. Section 2 introduces the application scenario of spectrum sensing. Section 3 shows the method of processing spectrum sensing with information geometry. Section 4 mainly introduces deep neural networks. Section 5 is the experimental simulation. Section 6 is a summary of the paper.

## 2. Spectrum Sensing Model and Centralized Spectrum Sensing Scene

**Figure 1:**
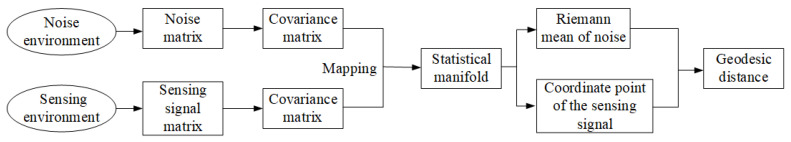
Spectrum sensing model.

The SUs sense noise in a noisy environment and calculate a covariance matrix of the noise matrix. Then, the information geometry method is used to map the covariance matrix to the manifold, and the Riemann mean of the noise on the manifold is calculated. Similarly, the signal covariance matrix extracted from the sensing environment is mapped onto the manifold. The geodesic distance of the sensing signal and Riemann mean is calculated, that is the statistical characteristics of the signal are also the input data of the network. The whole process can be seen in Figure 1.

**Figure 2:**
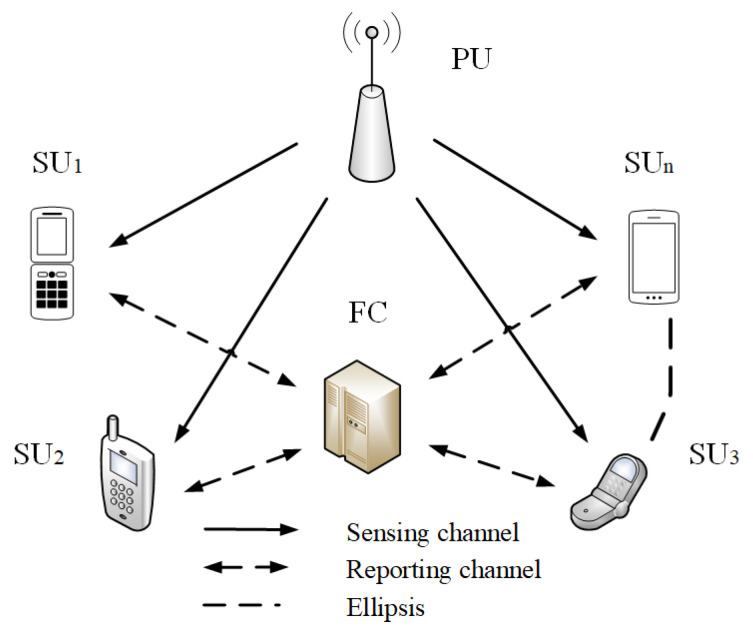
Centralized cooperative spectrum sensing. FC, fusion center.

In cognitive radio networks (CRNs), cooperative spectrum sensing (CSS) can not only overcome the physical limitations of single node spectrum sensing, but also achieve fast, accurate, and reliable sensing performance in the low SNR environments [22]. Cooperative spectrum sensing can be divided into three types: Distributed cooperative spectrum sensing, centralized cooperative spectrum sensing, and relay cooperative spectrum sensing. The paper uses centralized spectrum sensing, as shown in Figure 2. The centralized spectrum sensing system has a fusion center (FC). Each SU does not need to exchange the sensing information, but sends the decision data to the FC. The FC performs a fusion decision on the received information according to the decision criteria.

Suppose that there are *M* secondary users in the CRNs and the number of sampling points per SU is *N*. $H_{0}$ indicates that the PU signal does not exist, and $H_{1}$ indicates that the PU signal exists. Therefore, the sensing signal under the two assumptions can be expressed as:

(1) $$x_{m}\left(n\right)=\left\{\begin{array}{cc} w_{m}\left(n\right), & H_{0}\\ s_{m}\left(n\right)+w_{m}\left(n\right), & H_{1} \end{array}\right.,$$

m=1,2,⋯,M and n=1,2,⋯,N, where $s_{m}\left(n\right)$ represents the PU signal and $w_{m}\left(n\right)$ represents the Gaussian white noise signal. False alarm probability and detection probability can be defined as:

(2) $$P_{fa}=P\left[H_{1}|H_{0}\right],$$

(3) $$P_{d}=\left[H_{1}|H_{1}\right].$$

The signal sensed by SU constitutes a vector matrix $X={\left[x_{1},x_{2},\ldots ,x_{M}\right]}^{T}$, where $x_{m}=\left[x_{m}\left(1\right),x_{m}\left(2\right),\ldots ,x_{m}\left(N\right)\right]$ represents the signal sample value of the $m^{\mathrm{th}}$ SU. Therefore, the dimension of matrix X is M×N:

(4) $$\begin{array}{cc} X & ={\left[x_{1},x_{2},\ldots ,x_{M}\right]}^{T}\\ \; & =\left[\begin{array}{cccc} x_{1}\left(1\right) & x_{1}\left(2\right) & \cdots & x_{1}\left(N\right)\\ x_{2}\left(1\right) & x_{2}\left(2\right) & \cdots & x_{2}\left(N\right)\\ \vdots & \vdots & \ddots & \vdots \\ x_{M}\left(1\right) & x_{M}\left(2\right) & \cdots & x_{M}\left(N\right) \end{array}\right]. \end{array}$$

Thus, the statistical covariance matrix of the sensing signal:

(5) $$R=\frac{1}{N}{\mathrm{XX}}^{T}=R_{S}+R_{W},$$

where $R_{S}$ and $R_{W}$ represent the covariance matrix of the noise and signal, respectively. The probability density function given an γ-dimensional vector subject to a zero-mean Gaussian distribution is:

(6) $$p\left(x|R\right)=\frac{1}{\sqrt{{\left(2\pi \right)}^{\gamma }\det R}}\exp\left(-\frac{1}{2}x^{T}R^{-1}x\right),$$

where R is the ϖ-dimensional covariance matrix. The parameter model composed of the parameterized probability distribution family can be expressed as $S=\left\{p\left(x|R\right)|R\in C{}^{\varpi \times \varpi }\right\}$, where $C{}^{\varpi \times \varpi }$ is an open set in the ϖ-dimensional vector space. In information geometry, the structure of *S* is a microscopic manifold, also called a statistical manifold, where R is the coordinate point on the statistical manifold. Then, both $R_{W}$ and $R_{S}+R_{W}$ under the two hypotheses can map the sensing signals to the statistical manifolds as coordinates on the manifold.

## 3. Spectrum Sensing Based on Information Geometry

The mapping between the sampled signal and the manifold was clarified in the previous section. Next, the calculation method of the two point distance on the statistical manifold will be introduced. Here, the geodesic distance is used to measure the distance between two points to reflect the characteristics of the signal.

### 3.1. Statistical Manifold and Fisher Metric

The parameterized probability density of a statistical manifold in an information geometry can be expressed as [23]:

(7) $$S=\{p\left(x;\xi \right)|\xi \in \Theta \subset C^{\varpi \times \varpi }\},$$

where the vector in the parameter space obeys a multivariate Gaussian distribution with a mean of zero. In $\xi ={\left(\mu ,\sigma \right)}^{T}$, μ is the mean and $\sigma^{2}$ is the variance. In the literature [24], *S* is regarded as a statistical manifold with a dimension of ϖ. Each probability density p(x;ξ) is a point in S where the coordinates are ξ. Therefore, the covariance matrix of the sensing signal in spectrum sensing can be mapped to the manifold. The difference between the sensing signals can be measured by the distance between the two points on the statistical manifold. For the statistical manifold *S*, the Fisher information matrix plays the role of the Riemann metric, called the Fisher metric. Given the point p(x;ξ)∈S, the Fisher metric $G\left(\xi \right)=\left[g_{ab}\left(\xi \right)\right]$ at P on the manifold *S* is:

(8) $$g_{ab}\left(\xi \right)=E_{\xi }\left[\frac{\partial }{\partial \xi^{a}}\ln p\left(x;\xi \right)\frac{\partial }{\partial \xi^{b}}\ln p\left(x;\xi \right)\right],$$

where $E_{\xi }$ represents the mathematical expectation of p(x;ξ). Therefore, we calculate the distance between two points on the statistical manifold according to Equation (8).

### 3.2. Geodesic Distance and Riemann Mean

On the manifold *S*, assume that the arbitrary curve between the two points $\theta_{1}$ and $\theta_{2}$ is θ(t), $t_{1}\leq t\leq t_{2}$, $\theta \left(t_{1}\right)=\theta_{1}$, $\theta \left(t_{2}\right)=\theta_{2}$. Thus, the distance between $\theta_{1}$ and $\theta_{2}$ on curve θ(t) is [25]:

(9) $$D\left(\theta_{1},\theta_{2}\right)=\int_{t_{1}}^{t_{2}}\sqrt{{\left(\frac{d\theta }{dt}\right)}^{T}G\left(\theta \right)\left(\frac{d\theta }{dt}\right)dt},$$

where G(θ) represents $G\left(\xi \right)=\left[g_{ab}\left(\xi \right)\right]$ in Equation (8). It can be seen from the above equation that the distance between $\theta_{1}$ and $\theta_{2}$ is related to curve θ(t). When the minimum distance is obtained by Equation (9), the curve is called the geodesic line, and the minimum distance is called the geodesic distance. The signal sensed by SU is a multivariate Gaussian distribution family with the same mean, but different covariance matrices. Therefore, for two arbitrary points $Q_{1}$ and $Q_{2}$ on *S*, we can calculate the geodesic distance between $Q_{1}$ and $Q_{2}$ [26]:

(10) $$\begin{array}{cc} D(Q_{1},Q_{2}) & =\|\log(Q_{1}^{-1/2}Q_{2}{Q_{1}}^{-1/2}){\|}_{F}\\ \; & =\sqrt{\Sigma_{\nu =1}^{n}\log^{2}\left(\lambda_{\nu }\right)}, \end{array}$$

where $\lambda_{\nu }$ is the νth eigenvalue of $Q_{1}^{-1/2}Q_{2}{Q_{1}}^{-1/2}$.

For the *L* points $Q_{l}\left(l=1,2,\ldots ,L\right)$ in the statistical manifold, when *L* is two, the Riemann mean $\bar{Q}$ is located at the midpoint connecting the $Q_{1}$ and $Q_{2}$ geodesics, whose value is [27]:

(11) $$\bar{Q}=Q_{1}^{1/2}{\left(Q_{1}^{-1/2}Q_{2}{Q_{1}}^{-1/2}\right)}^{1/2}Q_{1}^{1/2}.$$

For the case of L>2, the Riemann mean $\bar{Q}$ is difficult to find directly. According to the gradient descent algorithm in [28,29], the iterative calculation method of $\bar{Q}$ can be derived, so the expression of the Riemann mean can be obtained as:

(12) $${\bar{Q}}_{\alpha +1}={{\bar{Q}}_{\alpha }}^{1/2}\exp\left(\frac{\tau }{L}\Sigma_{l=1}^{L}\log({{\bar{Q}}_{\alpha }}^{-1/2}Q_{l}{{\bar{Q}}_{\alpha }}^{-1/2})\right){{\bar{Q}}_{\alpha }}^{1/2},$$

where τ is the iteration step size with 0≤τ≤1, α is the number of iteration steps, and *l* is the ordinal number of the point.

We can calculate the Riemann mean of the noise in the noisy environment by Equation (12). Then, we calculate the geodesic distance of the signal mapped to the manifold to the Riemann mean according to Equation (10). Finally, the geodesic distance is the input data for the neural network.

## 4. Spectrum Sensing Based on the Deep Neural Network

**Figure 3:**
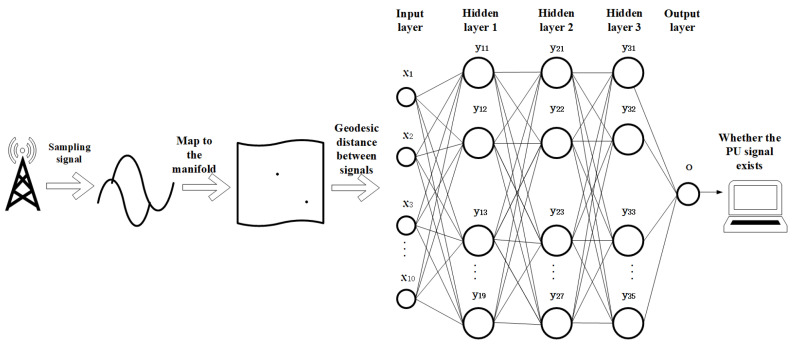
The learning model of deep neural network.

The whole training process is shown in Figure 3. The SU is greatly affected by SNR when performing signal detection. Low SNR will result in low detection performance of the system. Therefore, the input data of deep neural network are the detection result of multiple SUs under different SNRs.

### 4.1. Determination of the Dataset

First, multiple SUs sense the energy value of only the presence of noise and the signal doped with noise under different SNR conditions. Then, with the same SNR, the geodesic distance between the noise and the sensing signal is calculated. Finally, the dataset composed of the geodesic distance is used as the input data of deep neural network. This paper divides the input data into three parts: Training dataset, validation dataset, and test dataset. The training dataset is used to train the neural network. The validation dataset is used to select the hyperparameters of the neural network. The test dataset is used to test the classification ability of the network.

### 4.2. Selection of Hyperparameters in the Deep Neural Network

The selected structure of deep neural network is composed of an input layer, an output layer, and three hidden layers.

Input layer: Take the geodesic distance between the signals extracted by the information geometry method as input data. We select the sensing signals at 10 different SNRs; thus, the input layer selects 10 neurons.

Output layer: The output of deep neural network is to determine whether the PU signal exists, so we set the output layer as a neuron.

Hidden layer: We use a neural network with three hidden layers. In SNR = −18 dB∼SNR = −12 dB, two SUs sense 10 times every 0.01 SNR. Then, the geodesic distance between the sensing signals is calculated at the SNR. The input data thus obtained are 1200 sets, and each set of features is 11. The 11th column represents the classification label. The first 1000 groups in the input data are used as training data; 1001∼1100 groups are used as verification data; and 1101∼1200 groups are used as test data. Table 1 was obtained by simulation using the validation dataset. As shown in Table 1, by comparing the accuracy of different hidden layers, it is shown that the network with three hidden layers has the highest accuracy.

Similarly, the number of neurons in the three hidden layers in the depth model was verified with the verification data, which were 9, 7, and 5, respectively. The experimental results are shown in Table 2.

As shown in Table 3, by comparing the two activation functions of Sigmoid and Tanh, the activation function of this model is set as the Tanh function.

Given two probability distributions *p* and *q*, the cross-entropy can be defined as:

(13) $$H\left(p,q\right)=-\Sigma_{i=1}^{n}p\left(c_{i}\right)\cdot \log(q\left(c_{i}\right)),$$

where $p(c_{i})$ is the true distribution for the *i*th data, $q(c_{i})$ is the theoretical distribution of the neural network, and n is the number of samples.

### 4.3. Training of the Deep Neural Network

In this paper, the geodesic distance between signals is used as the input data. The training sample set is $\left[c_{1},c_{2},\ldots ,c_{z}\right]$, and *z* is the number of training samples. Any one of the samples $c_{f}=\left[c_{f1},c_{f2},\ldots ,c_{fW}\right]$, and *W* is the number of features. The geodesic distance is between the sensing signal and the noise Riemann mean. If the PU is present, set the target output value to one; else, set it to zero for training. Then, the target output value is $\left[d_{1},d_{2},\ldots ,d_{z}\right]$, and any one of the target output values *d* is one or zero. The actual output value is $\left[0_{1},0_{2},\ldots ,0_{z}\right]$, and the expected error is $e_{0}$. The error value after the *k*th iteration satisfies $e\left(k\right)<e_{0}$, $e\left(k\right)=\left|d(k)-o(k)\right|$.

### 4.4. Testing of the Deep Neural Network

After the network is trained, the test sample set $\left[c_{s+1},c_{s+2},\ldots ,c_{Z}\right]$ is placed as input data in deep neural network for training. The actual output of the test sample set is $\left[o_{s+1},o_{s+2},\ldots ,o_{Z}\right]$. Finally, it is judged whether the PU signal exists according to whether the data value is close to zero or one.

### 4.5. The Training Process of the Feedforward Neural Network

The learning rate is set to *v*, and the weight value between the previous layer neuron *i* and the current layer neuron *j* in the network model is $w_{ij}$.

When the network model is training forward, the input values of the hidden layer and the output layer are:

(14) $$I_{j}=\Sigma_{i}w_{ij}O_{i}+\theta_{j},$$

where $O_{i}$ means the output value of neurons *i* from the previous layer. $\theta_{j}$ represents the bias term of neurons from the current layer. The output of neurons from the current layer is related to the activation function. Therefore, the output value of the neuron from the layer is:

(15) $$O_{j}=\frac{1}{1+e^{-I_{j}}}.$$

When the network model is training in the opposite direction, the value of the error for the output layer:

(16) $$e_{j}=O_{j}\left(1-O_{j}\right)\left(d_{j}-O_{j}\right),$$

where $d_{j}$ is the label value of the neuron, and the value of the error for the hidden layer:

(17) $$e_{j}=O_{j}\left(1-O_{j}\right)\Sigma_{h}e_{h}w_{hj},$$

where $w_{hj}$ is the weight of the neuron *h* from the higher hidden layer to the neuron *j* from the current hidden layer. $e_{h}$ stands for the error of the neuron *h* from the higher hidden layer.

The training process of Feedforward neural network is shown in Algorithm 1.
**Algorithm 1:** Classification learning algorithm.**Initialization:**Weight values and bias terms for deep neural networks.**Step 1:****While**$e\geq e_{0}$ do**Step 2:** **for** Training any sample *c* in sample set $\left[c_{1},c_{2},\ldots ,c_{z}\right]$ do  **Forward training****Step 3:**  **for** Each neuron *j* in the hidden or output layer, do**Step 4:**$I_{j}=\Sigma_{i}w_{ij}O_{i}+\theta_{j}$,
$O_{j}=\frac{1}{1+e^{-I_{j}}}$**Step 5:**  **End for**  **Reverse training****Step 6:**  **for** Each neuron *j* in the output layer, do**Step 7:**$e_{j}=O_{j}\left(1-O_{j}\right)\left(d_{j}-O_{j}\right)$**Step 8:**  **End for****Step 9:**  **for** In the training process from the last hidden layer to the first hidden layer,  each neuron *j* of the hidden layer, do**Step 10:**$e_{j}=O_{j}\left(1-O_{j}\right)\Sigma_{h}e_{h}w_{hj}$**Step 11:**  **End for****Step 12:**  **for** Every deviation $\theta_{j}$ in the network model, do**Step 13:**$\Delta \theta_{j}=\left(l\right)e_{j}$,
$\theta_{j}=\theta_{j}+\Delta \theta_{j}$**Step 14:**  **End for****Step 15:** **End for****Step 16:****End while**

## 5. Simulation Experiment and Result Analysis

The spectrum sensing method combining information geometry and deep neural network was simulated by MATLAB. There are multiple signals in the actual environment, so this paper sets up a multi-signal scenario to verify the performance of the proposed algorithm.

### 5.1. Multi-Component Signal

The PU signal was a multi-component signal $S\left(t\right)=cos\left(t\right)+cos\left(4t+0.2t^{2}\right)$, and the noise was ideal Gaussian white noise.

**Figure 4:**
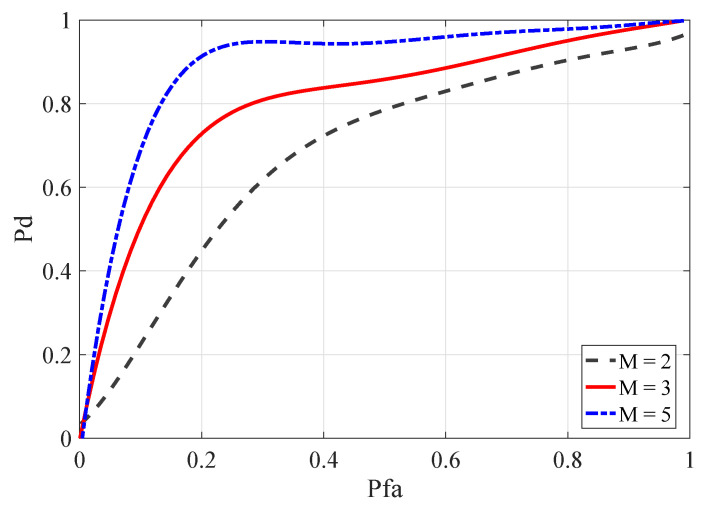
ROC curves for the number of SUs in different cases.

The number of SUs had a large impact on the sensing effect. In order to verify the influence of the number *M* of SUs on the detection performance, the number *M* of SUs was set to 2, 3, and 5 in the range of SNR = −21 dB∼SNR = −15 dB. Similarly, we trained the first 1000 groups of input data as training data and then used the latter 200 samples as test data to test the network performance. In Figure 4, Pd represents the detection probability, and Pfa represents the false alarm probability. It can be seen from the figure that when the false alarm probability is the same, as the number *M* of SU increases, the sensing performance of the method also steadily increases.

**Figure 5:**
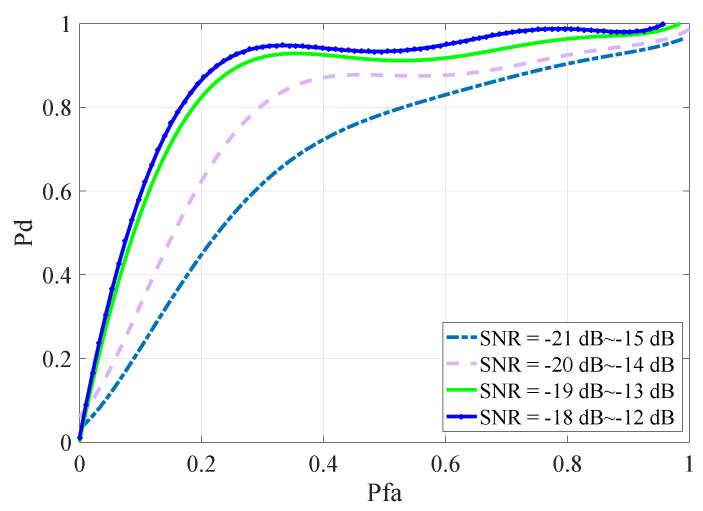
ROC curves at different SNRs.

SNR was also one of the factors affecting the sensing performance of the algorithm. In order to explore the influence of SNR on the proposed algorithm, SNR was set to different value intervals, and the SUs were two. The same method was used to simulate the data extraction. It can be seen from Figure 5 that where the false alarm probability is the same, the sensing signal performance of the neural network is better in the interval where SNR is larger.

**Figure 6:**
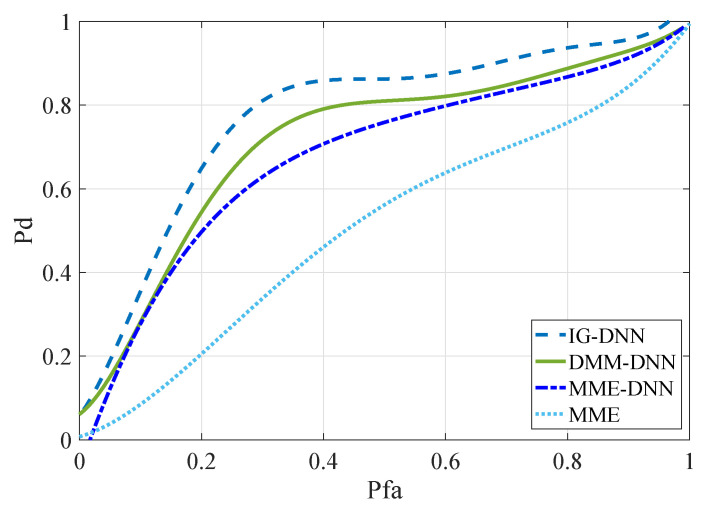
ROC curves for different methods with the same SNR (a).

In order to verify the superiority of the information geometry and spectrum sensing method, the following comparisons were made under the same SNR conditions, as shown in Figure 6. In SNR = −20 dB∼SNR = −14 dB, IG-DNN, MME-DNN, and DMM-DNN calculated the sampling signal characteristics under the SNR, respectively. The MME determined the sensing signal by setting the decision threshold at a fixed SNR. Therefore, SNR = −14 dB, and the number of SUs was two for the simulation experiment. According to the ROC curves of MME and MME-DNN, it can be seen that when the false alarm probability were the same, the neural network algorithm had higher detection probability than the traditional classification algorithm. By comparing the IG-DNN algorithm with the other three methods, the application of information geometry to spectrum sensing improved the sensing performance. Inaccurate selection of threshold values can affect sensing performance, but deep neural network avoided setting decision thresholds.

**Figure 7:**
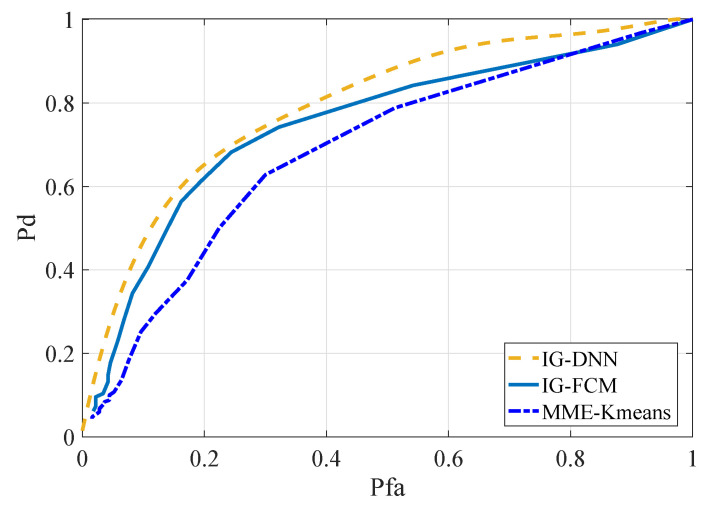
ROC curves for different methods with the same SNR (b).

Figure 7 compares the sensing algorithm proposed in this paper with other sensing algorithms. The IG-FCM algorithm was proposed in [17], and the MME-Kmeans algorithm was proposed in [30]. In the figure, IG-DNN is in the range of SNR = −20 dB∼SNR = −14 dB, and the other two methods are SNR = −17 dB for the simulation experiments. The number of SUs for the aforementioned methods was two. It can be observed from the ROC curve that the IG-DNN algorithm had the best sensing performance when the false alarm probability was the same.

### 5.2. AM Signal

The PU signal of the previous part was a multi-component signal, where the PU signal was replaced by an AM signal to further verify the IG-DNN algorithm. The AM signal was S(t)=(1+sin(2·pi·2000t))+cos(2·pi·fc·t), where *t = n/fs*, *fs* is the sampling frequency, and *fc* is the signal frequency.

**Figure 8:**
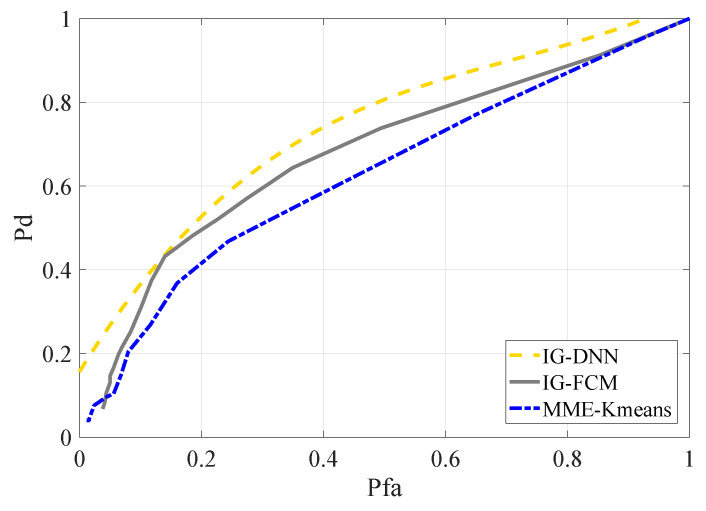
ROC curves for different methods in the AM signal scenario, SNR = −15 dB.

**Figure 9:**
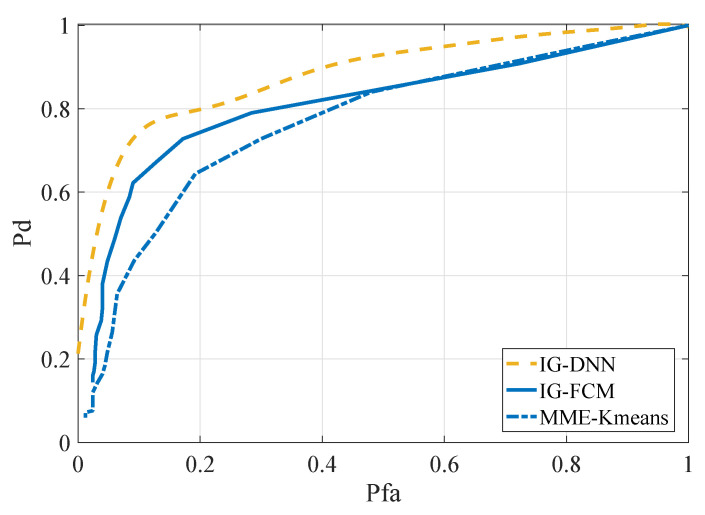
ROC curves for different methods in the AM signal scenario, SNR = −13 dB.

The IG-DNN algorithm was in the range of SNR = −18 dB∼SNR = −12 dB and SNR = −16 dB∼SNR = −10 dB, respectively. The other two methods were SNR = −15 dB and SNR = −13 dB, respectively. The number of SUs for the aforementioned methods was two. The simulation results are shown in Figure 8 and Figure 9. According to the simulation results of the two graphs, the performance of the proposed IG-DNN algorithm was better than the other two methods.

## 6. Conclusions

The IG-DNN algorithm proposed in this paper improved the sensing performance of the spectrum to some extent. The characteristics of the sensing signal were reflected in the statistical manifold, and the difference of the points on the manifold was calculated by the method of information geometry. Then, deep neural network was used to classify the geodesic distance to obtain the spectrum sensing classifier. Finally, the classifier was used to classify the unknown signals to determine whether the PU existed. Simulation experiments showed that the IG-DNN algorithm had better spectrum sensing performance. In future work, we are going to study array signal algorithms and a single sensor model. We will apply array signal algorithms or the single sensor model of spectrum sensing to further improve sensing performance.

## Figures and Tables

**Table 1:** Comparison of testing accuracy using different hidden layers.

Number of Experiments	1	2	3	4	5	6	7	8	9	10	Average Value
1 hidden layer (%)	88	92	89	92	89	93	94	90	92	87	90.6
2 hidden layer (%)	89	93	93	90	92	92	92	89	95	91	91.6
3 hidden layer (%)	92	93	89	94	94	91	93	91	92	93	92.2
4 hidden layer (%)	94	94	91	93	89	91	91	92	9	93	91.9

**Table 2:** Comparison of testing accuracy using different numbers of neurons.

Number of Experiments	1	2	3	4	5	6	7	8	9	10	Average Value
5/2/2 (%)	94	90	92	89	92	93	90	94	90	89	91.3
9/7/5 (%)	92	94	89	94	96	89	90	89	94	93	92.0
20/10/8 (%)	88	95	91	93	93	87	92	91	92	92	91.4

**Table 3:** Comparison of testing accuracy using different activation functions.

Number of Experiments	1	2	3	4	5	6	7	8	9	10	Average Value
Sigmoid(%)	95	91	89	92	92	88	95	88	91	89	91.0
Tanh(%)	95	95	96	90	94	93	88	94	92	89	92.6
